# Diagnostic Pitfalls of Macrocephaly and Intracranial Dural Arteriovenous Fistulas: Connecting the Dots With the Red Flags

**DOI:** 10.7759/cureus.55288

**Published:** 2024-02-29

**Authors:** Alina Andrei, Thomas Saliba, Boris Lubicz, Christophe Fricx

**Affiliations:** 1 Pediatric Neurology, University Hospital of Brussels (HUB) - Queen Fabiola Children's Hospital/Université Libre de Bruxelles (ULB), Brussels, BEL; 2 Radiology, University Hospital of Brussels (HUB) - Queen Fabiola Children's Hospital/Université Libre de Bruxelles (ULB), Brussels, BEL; 3 Interventional Neuroradiology, University Hospital of Brussels (HUB) - Erasme Hospital/Université Libre de Bruxelles (ULB), Brussels, BEL; 4 Pediatrics, University Hospital of Brussels (HUB) - Erasme Hospital/Université Libre de Bruxelles (ULB), Brussels, BEL

**Keywords:** cervical venous hum, pediatric intracranial vascular malformations, cerebral angiography, magnetic resonance arteriography, dural arteriovenous fistulas, hydrocephaly, head circumference, macrocephaly

## Abstract

Macrocephaly is defined as an abnormal increase in head circumference greater than two standard deviations above the mean for a given age and sex. We present the case of a 16-month-old boy with congenital progressive macrocephaly, who was referred to our hospital for a ventriculoperitoneal shunt placement for external hydrocephalus diagnosed at 13 months of age. The patient had a febrile seizure 12 hours after the shunt was placed and the emergency CT exam revealed collapsed ventricles and a right frontal subdural collection, suggestive of an over-drainage and intracranial hypotension. A subsequent electroencephalogram (EEG) revealed some anomalies, but the patient was discharged two days later due to having no neurological symptoms after being placed on anticonvulsants. The patient returned to the hospital one week later due to recurrent seizures. Further clinical examination revealed prominent and tortuous veins of the skull, palpated in the left occipital region. A thrill and a left carotid murmur were heard during auscultation. A subsequent brain MRI with MR arteriography and venography was performed in search of an explanation for hydrocephaly. The sequences were suggestive of a dural arteriovenous fistula, which was confirmed and then treated using coils during an interventional angiography. A second procedure was performed two months later to complete the embolization, with subsequent imaging follow-ups showing the procedure to have been successful. The measurement of the cranial circumference, its regular evaluation, and its evolution allow a hierarchical diagnosis strategy by distinguishing primary and secondary macrocephaly, progressive or not. Dural arteriovenous fistulas (DAVF) are an under-appreciated cause of macrocephaly, with which they are associated in 35% of cases. Intracranial DAVFs are pathologic shunts between dural arteries and dural venous sinuses, meningeal veins, or cortical veins. Patients with DAVFs may be completely asymptomatic. Symptoms, when present, may range from neurological deficits, seizures, and hydrocephaly to fatal hemorrhage. The symptoms depend on the location and venous and drainage patterns of the DAVF. They can be difficult to identify on routine MRIs unless specifically searched for, especially in cases of technically suboptimal examinations. We aim to give a practical approach to identify the clinical clues that warrant further investigation. Several specific protocols exist regarding the management of macrocephaly and should be followed carefully once a diagnosis has been reached, but further studies are needed to integrate more clinical and neuroimaging findings to permit an early diagnosis.

## Introduction

Macrocephaly is defined as an abnormal increase in head circumference greater than two standard deviations above the mean for a given age and sex. Macrocephaly affects up to 5% of the pediatric population [1], and it represents a relatively common presenting symptom in the pediatric population during routine well-child examinations [2]. Measuring the head circumference, its regular evaluation, and its evolution kinetics allows a hierarchical diagnosis strategy by distinguishing primary (congenital) or secondary (acquired) macrocephaly, progressive or not [1]. These characteristics are good instruments for recognizing the development of the skull and its contents (the brain, CSF spaces, and cerebral vascularization) [3].

The major diagnostic consideration when dealing with a case of macrocephaly is to distinguish between hydrocephaly (excessive volume of intracranial CSF), space-occupying lesions, and megalencephaly (enlargement of the brain) [4]. Clinically, the distinction between megalencephaly (enlarged brain) and macrocephaly (enlarged head overall) relies on neuroimaging examination of the brain and recognition of enlarged cerebral structures [5]. A patient’s clinical history and physical examination are crucial in the evaluation of macrocephaly, often determining the need for neuroimaging [6].

Family history should be taken to identify a possible genetic syndrome associated with macrocephaly. Furthermore, the parent's head circumference should always be measured, as isolated benign megalencephaly is a family trait in more than 50% of cases. An accurate developmental history should be taken to determine if the child has reached all developmental milestones and to evaluate any evidence of developmental regression and behavior change. Medical history should be aimed at detecting possible causes of hydrocephalus (e.g., prematurity, intraventricular hemorrhage, meningitis, intracranial neoplasm), presence of birth defects, neurological abnormalities, and skin and vascular abnormalities. The growth rate of head circumference should be carefully compared to previous values to look for any noticeable changes [1].

A careful physical exam should look at the presence of organomegaly (observed in overgrowth and metabolic disorders), skin and vascular anomalies (e.g., café-au-lait spots, hypopigmented macules, freckles, cutaneous naevi, hemangiomas, and other anomalies seen in neurocutaneous syndromes), craniofacial dysmorphisms, skeletal anomalies, and other congenital malformations [1].

In the context of macrocephaly, the following red flags must be kept in mind: tense or bulging fontanelle, flared cranial sutures, prominent superficial veins of the skull, rapidly progressive increase in head circumference, behavioral changes: irritability or drowsiness, asthenia, recently installed strabismus, palpebral ptosis, blurred or double vision, anisocoria or “sunset” gaze, nausea or vomiting, gait abnormalities, tremor, loss of consciousness or seizures, and papilledema [1,3,6-8].

Head circumference measurement is integrated into the routine care of healthy children: measured at birth and repeatedly for the first five years of life. Head circumference should be checked more frequently between 6 and 14 months, a period of sudden and rapid growth, or when macrocephaly has already been diagnosed [7]. An important red flag is when the infant is less than six months old and the head circumference increases by more than 2 cm in one month, which should lead to further testing [5].

The management of macrocephaly varies according to etiology. Neuroimaging, specifically magnetic resonance imaging (MRI), completed by MR arteriography and venography, is useful as a tool to confirm the clinical suspicion of an underlying developmental etiology [5-6].

## Case presentation

**Figure 1:**
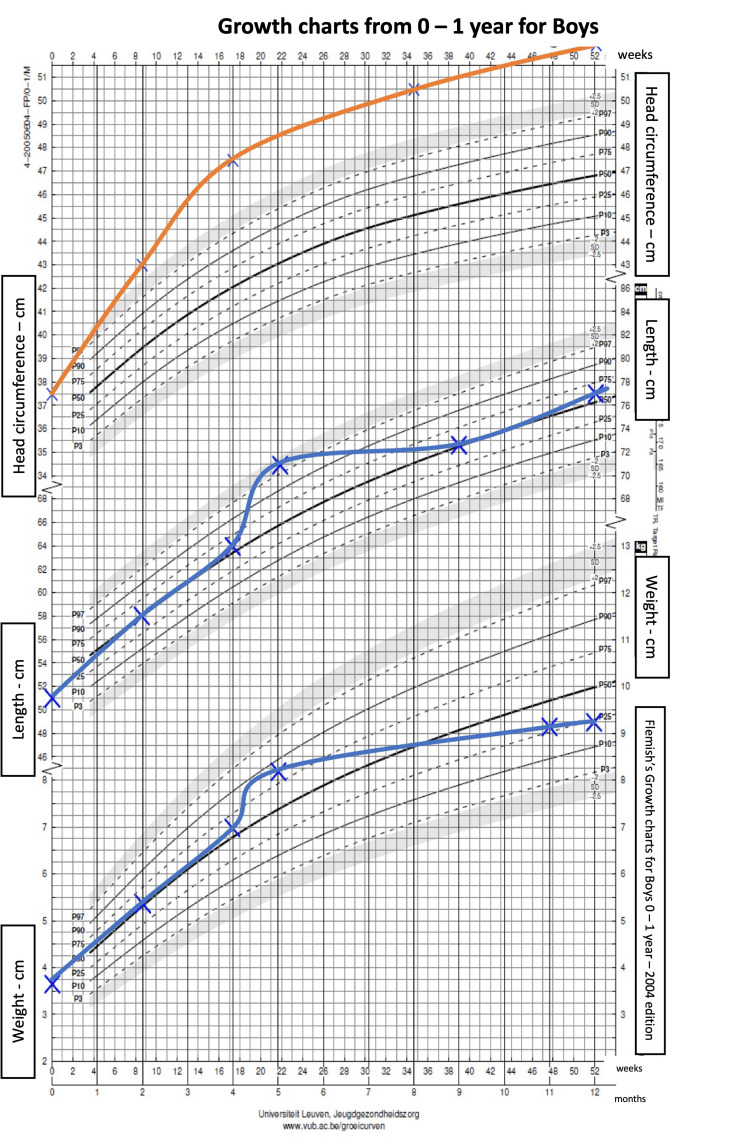
Patient’s growth charts from birth until 12 months A rapidly increasing lift was noticed in the first months of life, permitting the diagnosis of progressive macrocephaly before the age of six months. The patient's measurements (orange and blue lines) were plotted against the standard Belgian growth charts.

**Figure 2:**
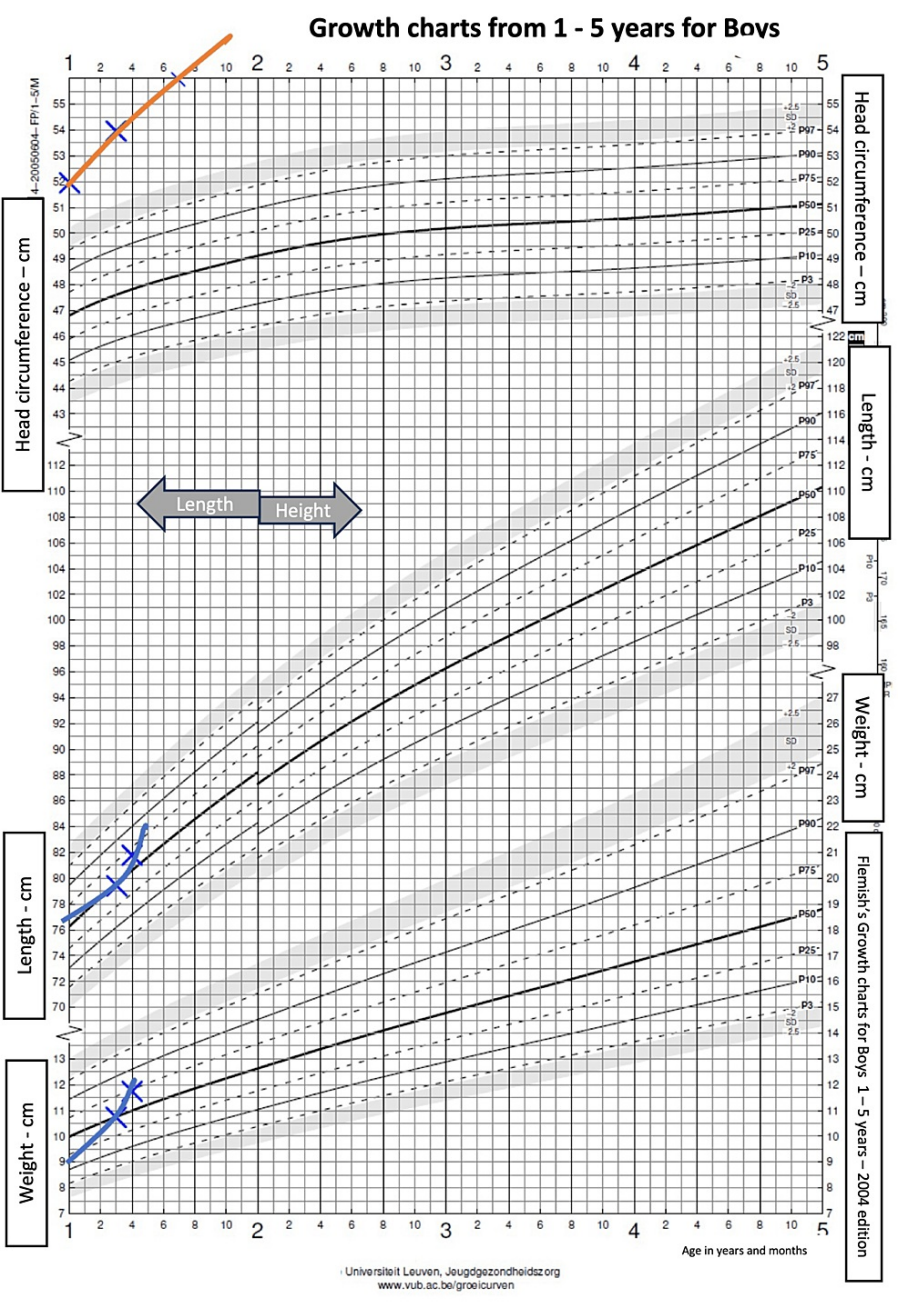
Patient’s growth charts from one to five years The patient’s measurements (orange and blue lines) were plotted against the standard Belgian growth charts.

A 16-month-old boy was referred to Erasme Hospital’s pediatric department for a scheduled surgical intervention and the installation of a ventriculoperitoneal shunt in the context of external hydrocephalus. He was born at term by emergency C-section for a narrow pelvis and failed forceps extraction, following an uneventful pregnancy. The patient’s reported family history was negative for known or suspected genetic disease, birth defects, malformation syndromes, chromosomal disorders, metabolic disorders, developmental delay, and consanguinity. At birth, the baby's head circumference had been measured at 37.5 cm (just above the 99th centile with birth weight on the 25th centile). The patient’s medical history was unremarkable, but the father had already noticed, after birth, a thrill in the posterior cervical region, which was trivialized by medical staff. Abnormal head circumference measurements had been obtained on several occasions since birth, showing congenital progressive macrocephaly (Figures 1, 2).

**Figure 3:**
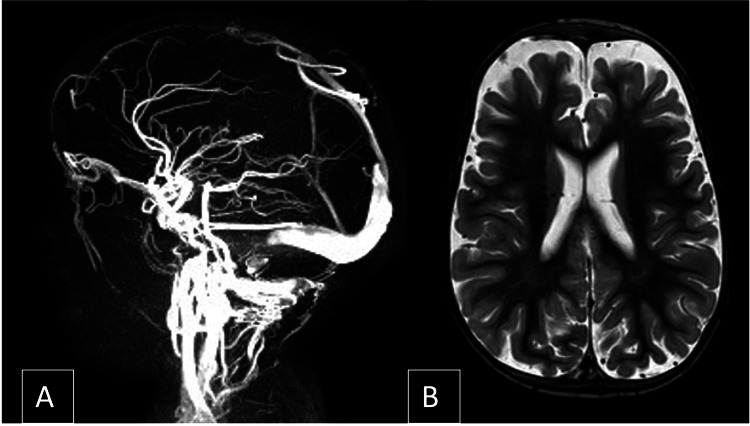
First brain MRI performed at 13 months old It was interpreted as being normal, apart from a dilated lateral sinus (A). The diagnosis of benign external hydrocephalus, a term used to define a rapid increase of head circumference in combination with increased subarachnoid space width (B), was made.

The head enlargement was first attributed to a benign form of familial macrocephaly. At 13 months of age, a brain MRI with MR venography (Figure 3) was performed because his head circumference was rapidly increasing. Apart from a dilated lateral sinus, it showed no other changes, leading to the diagnosis of external hydrocephaly being made.

**Figure 4:**
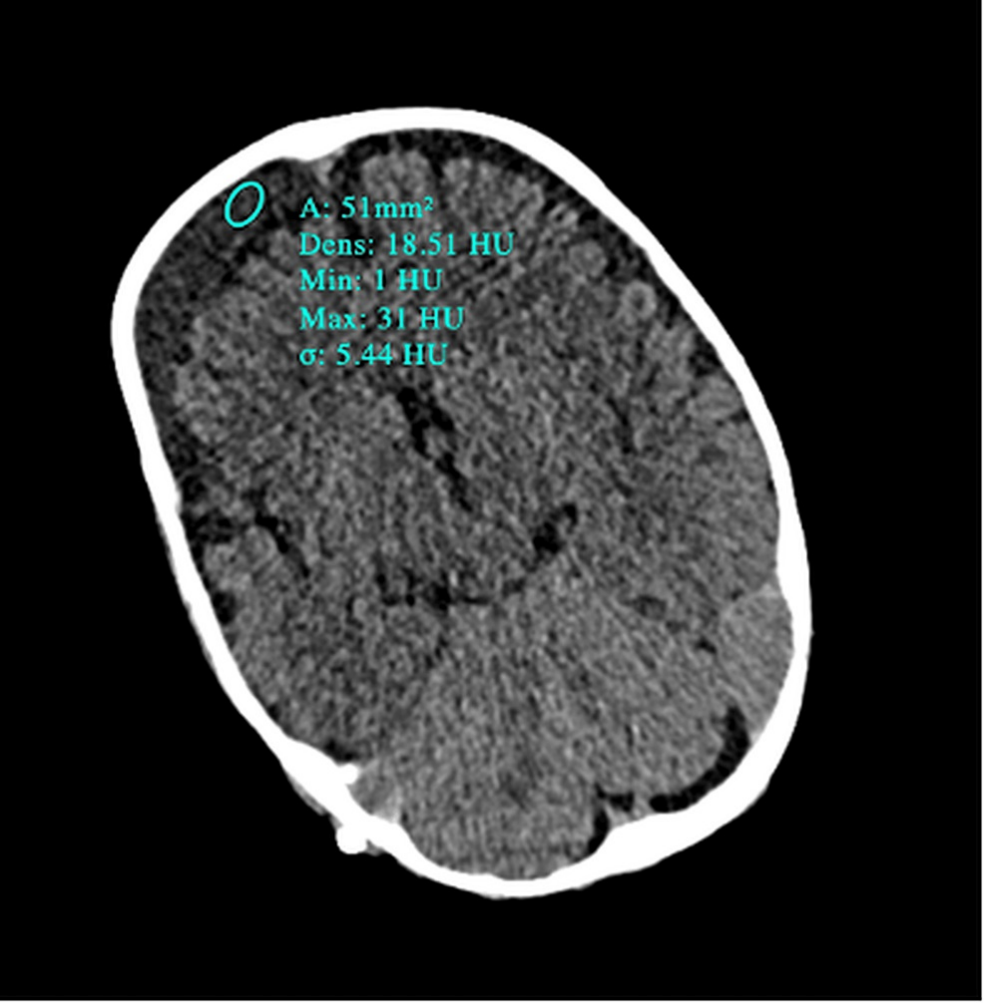
Brain scan performed 12 hours after ventriculoperitoneal shunting A partially collapsed ventricular system can be observed, particularly at the level of the third and fourth ventricles as well as the frontal horns of the lateral ventricles, suggesting over-drainage. A small subdural hypodense frontoparietal collection (maximum thickness of 21 mm) was found. It had a slightly higher density than the surrounding CSF, compatible with a subdural hygroma, most probably due to intracranial hypotension.

On the admission day, we found a normally developing boy, with a normal clinical and neurological examination and without any evidence of intracranial hypertension. His head circumference at presentation was 56 cm, well above the 99th centile, with a particular shape of his forehead showing prominent frontal bossing and prominent scalp veins. There was no history of vomiting or any other preceding illness in his medical history. His routine blood tests and cardiac evaluation (including an ECG and echocardiogram) revealed no abnormalities. A ventriculoperitoneal shunt (whose pressure was fixed at 5 cm H_2_O) was performed without periprocedural incidents reported. The patient presented with a generalized febrile seizure 12 hours after surgery and an urgent CT scan was performed, revealing a partially collapsed ventricular system and a right frontal subdural collection, suggestive of over-drainage and intracranial hypotension (Figure 4).

**Figure 5:**
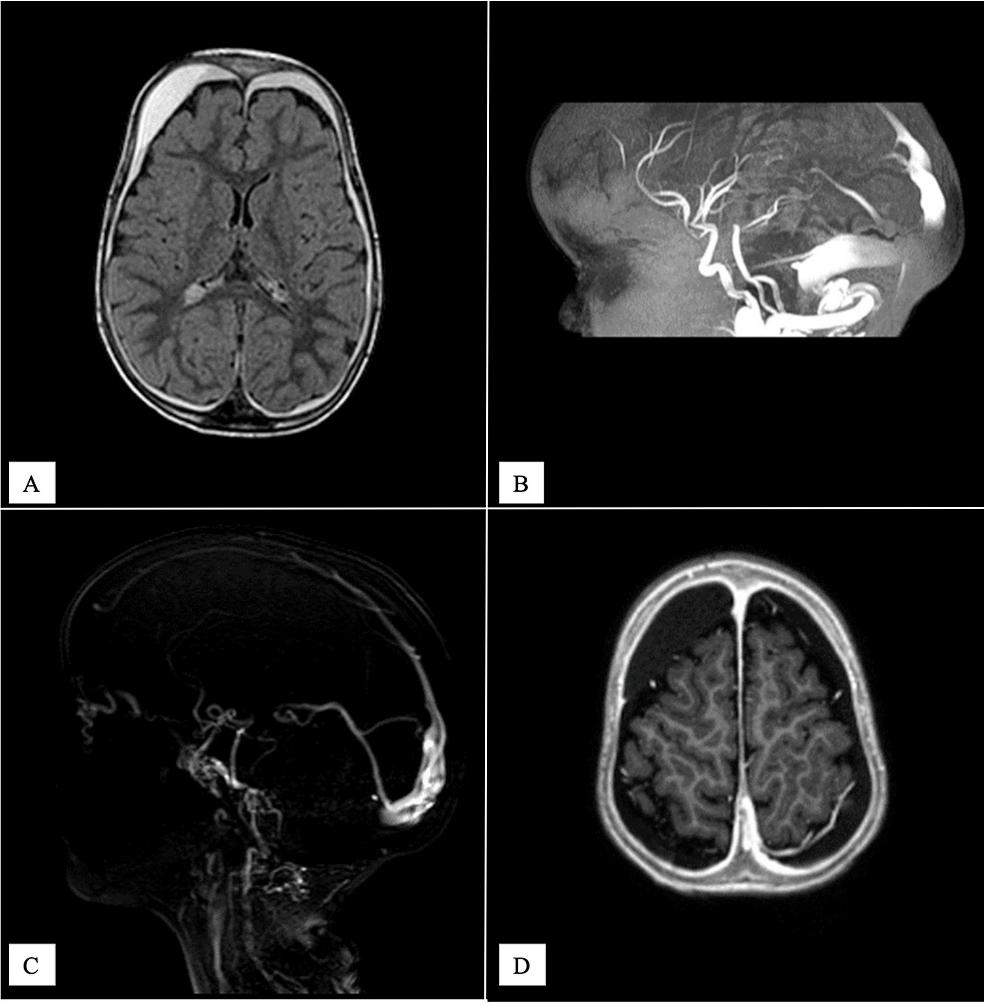
Brain MRI performed after ventriculoperitoneal shunting and seizures T2-weighted MRI (A) showing lateral ventricles of reduced size and bilateral subdural collections. This imagery is compatible with the diagnosis of intracranial hypotension due to overdrainage. Images B and C show MRI time of flight (TOF) imaging with enlargement of the transverse sinuses but without any sign of venous thrombosis. There is a communication between the left sigmoid sinus and the left occipital subcutaneous vascular network and arterialization of the left transverse and sigmoid sinuses as well as the posteroinferior part of the superior sagittal sinus. T1-weighted MRI (D) acquired after contrast injection showed diffuse meningeal enhancement compatible with intracranial hypotension.

An EEG was obtained, showing a right posterior slowdown with sharp waves in the right occipital lobe. An anticonvulsant treatment with levetiracetam 40 mg/kg/day was initiated and the valve was adjusted at 8 cmH_2_O. He was discharged two days after the procedure without any incidents (no vomiting, no headache, no seizure). The patient returned one week later due to a seizure relapse, justifying a dual therapy with the addition of carbamazepine (10 mg/kg/day) to control the seizures. A much more detailed and thorough clinical examination was carried out, revealing prominent and tortuous veins of the skull and palpation in the left region of the base of the skull with a thrill and a left carotid murmur heard during auscultation. A brain MRI was performed (Figure 5), raising the suspicion of an arteriovenous malformation between the left sigmoid sinus and external carotid artery, with multiple dilated cortical veins.

**Figure 6:**
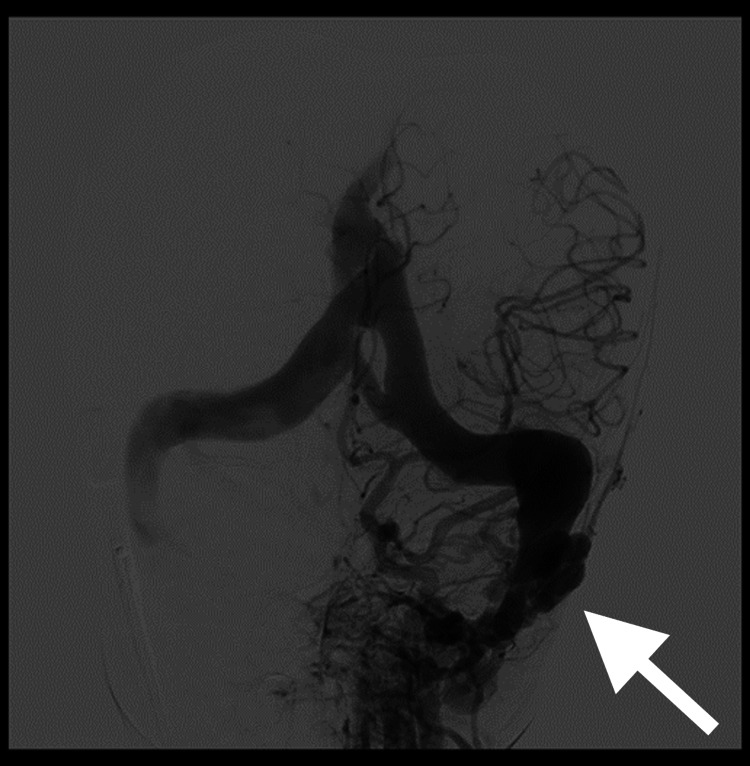
Angiographical image after contrast injection, which confirmed the diagnosis of a dural arteriovenous fistula between the left sigmoid sinus and a left occipital subcutaneous vascular network (arrow), a collateral artery from the external carotid artery The lesion was classified as Cognard type IIa+b, indicating a high risk of intracranial rebleeding and thus requiring early curative treatment.

Following this discovery, we performed an angiography (Figure 6), which confirmed the presence of a dural arteriovenous fistula of the left sigmoid sinus.

**Figure 7:**
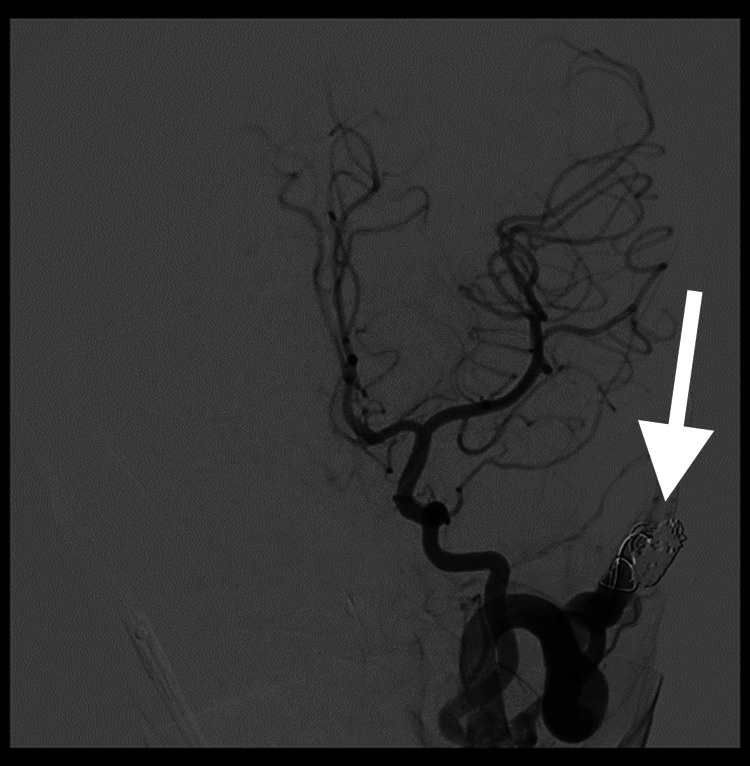
Post-intervention arteriography showing the stent (arrow) within the arteriovenous fistula, which nevertheless remained permeable as planned at the start of the procedure The cerebral blood flow was otherwise unremarkable.

He underwent emergency endovascular treatment by coiling achieving partial occlusion (Figure 7).

**Figure 8:**
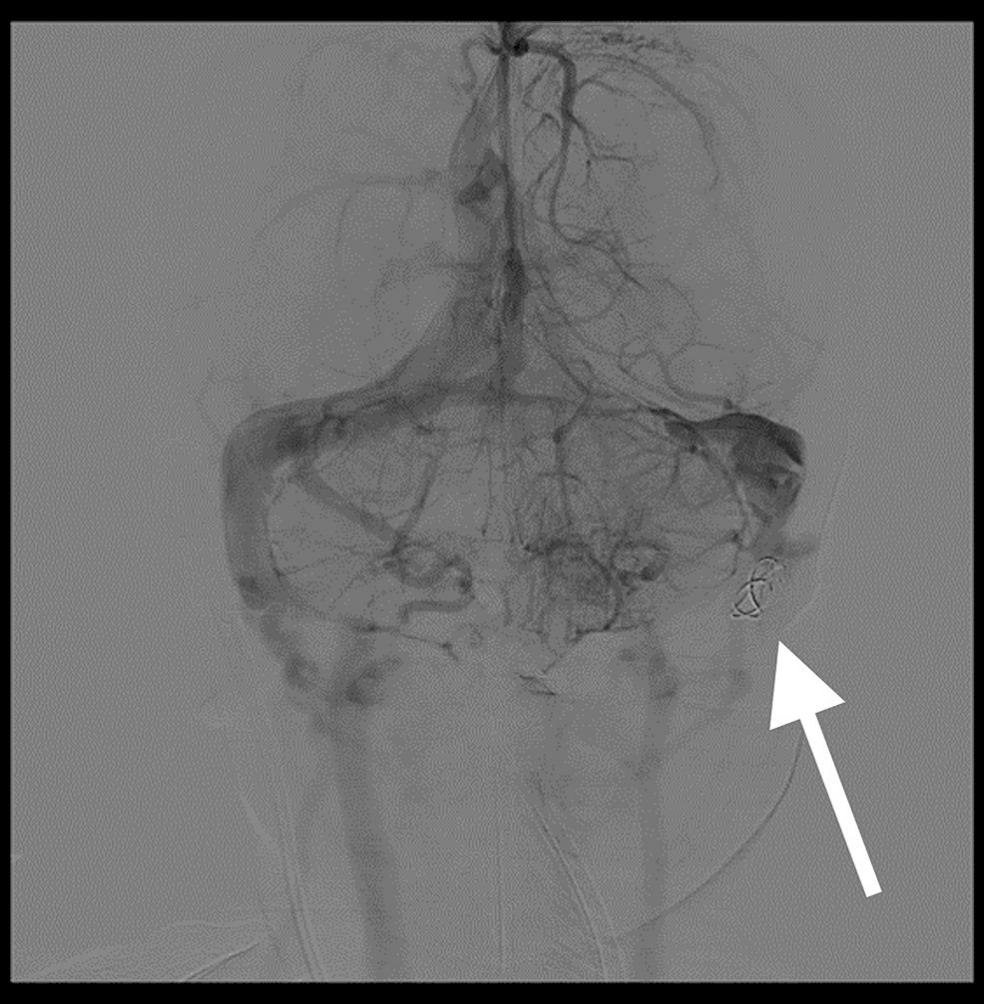
Arteriography showing a discreet “ballooning” of the left transverse sinus, which was part of the dural malformation This resulted in three more months of anticoagulant therapy with enoxaparin sodium in preventive doses to properly heal this area. The stent remained visible (arrow).

We have noticed a disappearance of the thrill and the left carotid murmur, and the valve of the ventriculoperitoneal shunt was blocked at 20 cmH_2_O to stop the CSF drainage. A prophylactic treatment with enoxaparin sodium was initiated after the intervention until the second session of embolization of the dural fistula was performed two months later. The second intervention made it possible to completely occlude the arteriovenous fistula (Figure 8) with perfect neurological tolerance post-procedure.

The postoperative course was uneventful. A cerebral MRI was performed after four months, revealing the absence of re-permeabilization of the fistula. However, persistent bilateral subdural collections were noted, slightly reduced in size, with the same enlargement of the lateral venous sinus, just as on the previous MRI. We also noted a reduction of the cortical venous dilatations, with the disappearance of signs of arterialization on the 3D time-of-flight (TOF) sequence of the left lateral venous and sigmoid sinus.

A withdrawal of ventriculoperitoneal shunt was performed 10 months after the initial surgery. The anticonvulsant treatment was gradually stopped because there were no more seizures and repeated EEGs did not show epileptiform discharges. He has been neurologically intact, and follow-up conventional angiography shows no recurrence of the dural arteriovenous fistulas at a one-year follow-up. Currently, the patient is three years old and his physical examination shows normal developmental milestones for his age and a normal neurological exam. The ophthalmological examination is also normal. He does not take any medication. His follow-up will consist of a brain MRI every two years initially.

## Discussion

**Figure 9:**
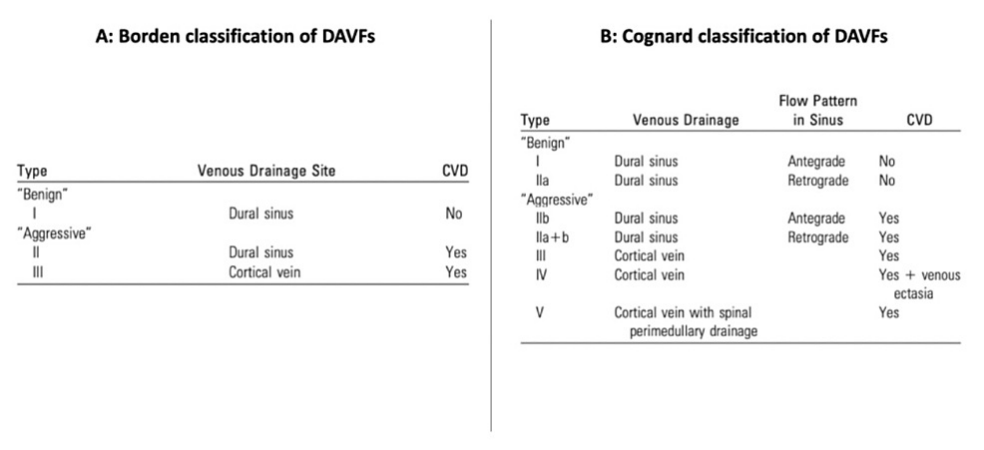
DAVFs classification (A and B) Lack of CVD (Borden type I, Cognard types I, IIa) is a favorable feature and is associated with a benign natural history. In either classification scheme, the presence of CVD (Borden type II and III, Cognard types IIb-V) is an aggressive feature that places DAVFs in a higher risk category due to increased hemorrhagic risk. Borden type I lesions have a direct communication of meningeal arteries with a meningeal vein or dural venous sinus and exhibit normal antegrade flow. Type II lesions have shunts between the meningeal arteries and the dural sinus, with retrograde flow into the subarachnoid veins, causing venous hypertension. Type III lesions have direct drainage of meningeal arteries into subarachnoid veins or an “isolated” sinus segment. The latter phenomenon is the result of thrombosis on either side of the arterialized sinus segment, which directs the retrograde flow into the subarachnoid venous system. Cognard classification (B) is based on the direction of dural sinus drainage, the presence or absence of CVD, and venous outflow architecture. Type I lesions drain into the dural sinus, have an antegrade flow direction, and lack CVD. Type II lesions are subdivided into three subcategories: type IIa lesions drain retrogradely into a dural sinus without CVD, type IIb lesions drain antegradely into a dural sinus with CVD, and type IIab lesions drain retrogradely into a dural sinus with CVD. Types III, IV, and V lesions all have CVD, absent dural venous drainage, and varying cortical venous outflow architecture. CVD - cortical venous drainage; DAVF - dural arteriovenous fistula

Dural arteriovenous fistulas (DAVFs) are rare vascular malformations usually presenting in infancy or childhood, representing a direct connection between dural arteries and cerebral venous sinuses and/or cortical veins [9-10]. The exact mechanism resulting in their formation remains unclear. The incidence and prevalence in childhood are unknown, and the most common clinical presentation in infants is congestive heart failure or it may be presented by an increased head circumference, focal neurologic deficits, or intracranial hemorrhage in children [10]. These fistulas are associated with hydrocephalus in 35% of cases in the pediatric population [11]. The DAVF venous drainage pattern determines the severity of symptoms and provides the foundation for the classification schemes (Figure 9) of Borden et al. and Cognard et al. Both of these systems associate cortical veinous drainage with an increased risk of intracranial hemorrhage and neurologic deficits [10].

The goal of treatment is occlusion of the arteriovenous fistula site or the feeding arteries and proximal draining vein as close as possible to the fistula [12-13]. The preferred treatment is embolization due to its lower mortality; however, it requires multiple interventions and in some cases, the lesion may recur. Early diagnosis is important to prevent neurological complications/sequelae and to reduce the morbidity and mortality due to intracranial hemorrhage [9].

Our patient had an unusual diagnostic process because the protocols were not followed completely and the red flags were not initially identified such as accelerated head circumference growth, prominent and tortuous veins of the scalp, cervical venous hum, and thrill at the palpation of the cervical region. These features have been described as part of the clinical picture of vascular intracranial malformation [11-12,14-17].

Furthermore, some false leads have led clinicians away from the final diagnosis: normal neurological examination, normal development, family history of benign essential macrocephaly, a significant growth spurt at 12 months old, with rapid growth in head circumference along with weight and height (a fact that is completely normal until the age of two years [7]). Although the initial MRI may have led to an earlier diagnosis, this was not the case since dural arteriovenous fistulas are very difficult to recognize on a non-contrast MRI and because the initial examination was of suboptimal technical quality. A unique aspect of his evolution was discovering a subdural collection immediately after draining the ventricular system, attributed to a subdural hygroma. Several possible mechanisms have been suspected to have caused the subdural collection. The most probable scenario was CSF over-drainage and secondary intracranial hypotension, though another possibility was effusion due to traumatized vessels of the dural fistula.

The presentation of this clinical case is a good example that highlights the importance of following the protocols and recognizing the red flags that influence diagnosis and prompt further investigations. Thanks to the fact that the patient started to have seizures after VP shunting, the decision tree was restarted from the beginning, and a detailed anamnesis and clinical examination were performed. The patient benefited from the necessary investigations to receive the correct diagnosis and the optimal treatment, having a favorable prognosis.

## Conclusions

Macrocephaly is a relatively common clinical condition affecting up to 5% of the pediatric population. There is no recognized standard classification system that integrates neuroimaging, clinical, molecular, genetic, and biological criteria for the management of children with macrocephaly. A tumoral cause must always be considered when dealing with a case of macrocephaly, and its exclusion should be the first and most important step in the evaluation pathway, in order to rule out raised intracranial hypertension, as it is a neurosurgical emergency. However, intracranial vascular malformations are an important, and likely common, cause of macrocephaly, especially in infants with intracranial bruits. It should therefore be considered in the diagnostic workup. If the macrocephaly is progressive, combined with signs of increased pressure in the venous system, such as pronounced veins on the scalp, cervical venous hum and thrill upon palpation, or signs of heart failure, we should proceed to further investigations such as magnetic resonance angiography or cerebral angiography. Future studies are required to correlate and integrate all the clinical data into practical approaches, in order to incorporate them into routine macrocephaly protocols and permit an early diagnosis and appropriate treatment.
